# Lesion Topography and Its Correlation With Etiology in Medullary Infarction: Analysis From a Multi-Center Stroke Study in China

**DOI:** 10.3389/fneur.2018.00813

**Published:** 2018-09-27

**Authors:** Yue-hui Hong, Li-xin Zhou, Ming Yao, Yi-cheng Zhu, Li-ying Cui, Jun Ni, Bin Peng

**Affiliations:** ^1^Department of Neurology, Peking Union Medical College Hospital, Chinese Academy of Medical Sciences, Beijing, China; ^2^Neuroscience Center, Chinese Academy of Medical Sciences, Beijing, China

**Keywords:** medullary infarction, lesion topography, lesion-etiology correlation, stroke mechanism, SMART

## Abstract

**Objectives:** The lesion topography of medullary infarction (MI) is heterogeneous and its correlation with stroke etiology remains elusive. We aim to clarify the lesion pattern of MI and to assess its correlation with stroke etiology.

**Material and Methods:** Of 1129 subjects with available DWI in SMART study (a multi-center trial concerning secondary stroke prevention in China) between April 2008 and December 2010, 43 patients with DWI confirmed MI (3.8%) were retrospectively evaluated. Lesions were categorized as lateral and medial medullary infarction (LMI and MMI, 33 and 10 subjects respectively) at 3 levels rostro-caudally and correlated with the stroke etiology. Clinical profiles and long-term prognosis were analyzed.

**Results:** Large artery atherosclerosis, small vessel occlusion, cardiogenic embolism and artery dissection accounted for 29, 11, 1, and 2 infarcts, respectively. Large artery disease was the most common cause in LMI (24 of 33, 72.7%) whereas small vessel occlusion was not uncommon in MMI (5 of 10, 50.0%). Though the difference of infarct pattern between large artery atherosclerosis and small vessel occlusion was insignificant, two distinct lesion patterns were considered to be relevant: (1) Rostral MMI with continuous medial pontine infarctions were more likely attributed to small vessel occlusion than large artery atherosclerosis. Kameda et al. (2) MMI with ventral to dorsal extension were more often caused by large artery disease than small vessel occlusion. Median NIHSS at admission was 4. During a median follow-up of 17 months, 2 patients died and 2 experienced recurrent ischemic events, 39 of 41 subjects (95.1%) were functional independent (mRS 0–2).

**Conclusions:** This multi-center study demonstrates that MI has distinct lesion pattern depending on various stroke etiologies and mechanisms. Future investigations with larger sample size should establish the lesion pattern of MI and validate its correlation with the stroke etiology and mechanisms, which might improve stroke management.

## Introduction

Medullary infarction (MI) is rare, and can be classified into lateral and medial medullary infarction (LMI and MMI) based on clinical and lesion patterns. LMI was more common than MMI and most lesions involved the rostro-middle medulla (1–4). Previous studies focused more on the anatomical, clinical, and topographical associations of MI (5–9). However, few correlated the lesion topography with the stroke etiology and mechanism of MI. Though the management in posterior circulation infarction remained uncharted in the past decades (10), it is important to distinguish the stroke etiology and mechanism since the treatment and prognosis may differ.

The arterial supply of medulla is distinct from that of the other brainstem areas. Briefly, they can be divided into four arterial groups (anteromedial, anterolateral, lateral, and posterior): (1) anteromedial and anterolateral groups arising from the vertebral and anterior spinal arteries. (2) lateral group arising from the posterior inferior cerebellar artery (PICA), the vertebral artery (VA), the basilar artery (BA), and anterior inferior cerebellar artery. (3) posterior group arising from the PICA for the rostral medulla and from the posterior spinal artery for the caudal medulla. The arterial territories have a variable extension at different levels. For example, the caudal regions of the medial medulla are supplied by paramedian branches of the anterior spinal artery, whereas more rostral located regions of the medial medulla are supplied by paramedian branches of the VA (9). Therefore, the mechanisms of MI might be different from that of other brain infarctions.

According to previous studies, the lesion patterns of MI were heterogeneous and its correlation with stroke etiology and mechanism was unclear. Distinct infarct patterns, if existed, would provide critical clues to accurate diagnosis. It was widely believed that VA atherosclerosis was the prominent cause of MI, through arterial embolism and/or branch occlusion (3, 11–13). However, some reports indicated that small vessel occlusion might be not uncommon in certain infarct patterns. Moreover, the hypoplasia/aplasia of VA is not uncommon and the branches of VA are poorly visualized via non-invasive angiography, making the distinction between large and small vessel occlusion rather difficult than that in anterior circulation infarction. This may explain the variable frequency of small vessel occlusion reported in previous studies (0–18.2%) (1, 4, 14). For example, small vessel occlusion might be the frequent etiology of rostral MI of anteromedial territory (32–63%) (3, 14–16) whereas MMI with dorsal extension or bilateral involvement (11) was more often related to large artery disease, probably by the way of branch occlusion. In the case of LMI, small infarct of dorsal (17) or lateral territory (18) might be associated with small vessel occlusion whereas large inferodorsolateral infarcts would indicate PICA occlusions (2). Despite the above implications, few were validated and disparities still existed (19, 20), therefore warrants further investigations.

Based on a multi-center database [SMART, Standard medical management in secondary prevention of ischemic stroke in China (21)], we sought to clarify the lesion pattern of MI. The lesion-etiology correlations based on angiography and lesion topography were also assessed, with particular emphasis on distinction between large artery atherosclerosis and small vessel occlusion.

## Methods

### Subjects

Data were collected from SMART, which was a multicenter, randomized controlled trial to evaluate the feasibility and efficacy of a guideline-based program in secondary stroke prevention across 47 hospitals in mainland China. A detailed description of study protocol and main results of SMART have been published elsewhere (21, 22). In brief, the participants were randomized to either a structured care group or a usual care group for secondary stroke prevention. The measure of intervention of SMART was in line with the current guideline of secondary stroke prevention. Among 3821 patients with acute ischemic stroke or transient ischemic attack (TIA) in the SMART study between April 2008 and December 2010, 1129 patients with available diffusion-weighted images (DWI) data were further evaluated. A sample of 43 patients from 20 centers with MI (3.8%) proven by DWI was enrolled in this subgroup analysis (13 subjects in structured care group and 30 in usual care group). The median time from symptom onset to DWI was 3 days (IQR 2–5 days). 33 patients had LMI (2.9%) and 10 had MMI (0.9%). The following clinical information was reviewed from database: age, gender and stroke risk factors [i.e., hypertension, diabetes mellitus, hypercholesterolemia, emboligenic heart disease, and smoking status. Definitions of risk factors were published in previous work (23, 24)]. To determine the neurological deficits and functional outcome, National Institutes of Health Stroke Scale (NIHSS) at admission and modified Rankin Scale (mRS) at discharge and follow-up were reviewed. The outcome was further categorized as favorable when the patients remained independent (mRS 0–2) and poor when dependent (mRS 3–6).

Vascular status was evaluated by magnetic resonance angiography (MRA), extracranial carotid and transcranial Doppler and axial T1 and T2-weighed MRI showing cross-sectional VA and BA. Computed tomography angiography (CTA) or digital subtraction angiography (DSA) was performed when arterial dissection was suspected. Emboligenic heart disease was detected by 12-lead electrocardiography and transthoracic echocardiography.

The SMART study was performed with the approval of the central ethics committee at the leading study center at Peking Union Medical College Hospital and of the ethics committees at all participating sites. Written informed consents were obtained from all participants or their legal surrogates.

### Classification of stroke etiology

The stroke etiologies were classified according to the modified TOAST (Trial of Org 10172 in acute stroke treatment) applied in previous studies (3, 25): (1) Large artery atherosclerosis (LAA) was defined as significant (>50%) stenosis at the relevant distal or proximal VA or proximal BA. (2) Cardiogenic embolism was diagnosed when emboligenic heart disease was detected according to TOAST criteria. (3) Small vessel occlusion (SVO) was defined when the infarction corresponded to the penetrator territory, and the patients had normal angiographic findings without emboligenic heart disease. (4) Arterial dissection was determined when the patient presented with concurrent neck or occipital pain and typical imaging features such as pearl and string sign, tapered stenosis or occlusion, intimal flap, double lumen, fusiform aneurysm, or intramural hematoma. VA hypoplasia detected by carotid ultrasound and/or MRA were defined if an asymmetry ratio of ≤ 1:1.7 of both VAs (26) and/or VA diameter ≤ 2.5 mm (27) was present.

### Assessment of lesion topography

**Figure 1:**
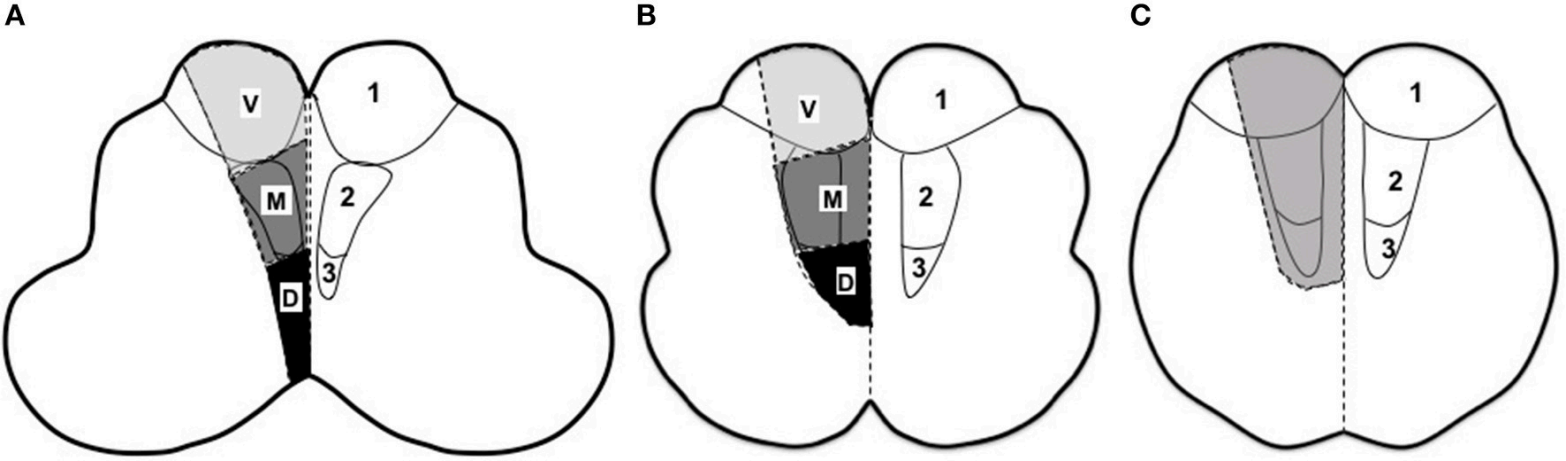
Rostro-caudal and horizontal division of medulla (11, 16, 28, 29). **(A)** Rostral medulla, characterized by dorsolateral bulging of the restiform body. **(B)** Middle medulla, characterized by the ventral-lateral bulging of the inferior olivary nucleus. **(C)** Caudal medulla, characterized by its round shape with closed fourth ventricle. V, ventral part; M, middle part; D, dorsal part. 1, corticospinal tract; 2, medial lemniscus; 3, medial longitudinal fasciculus.

**Figure 2:**
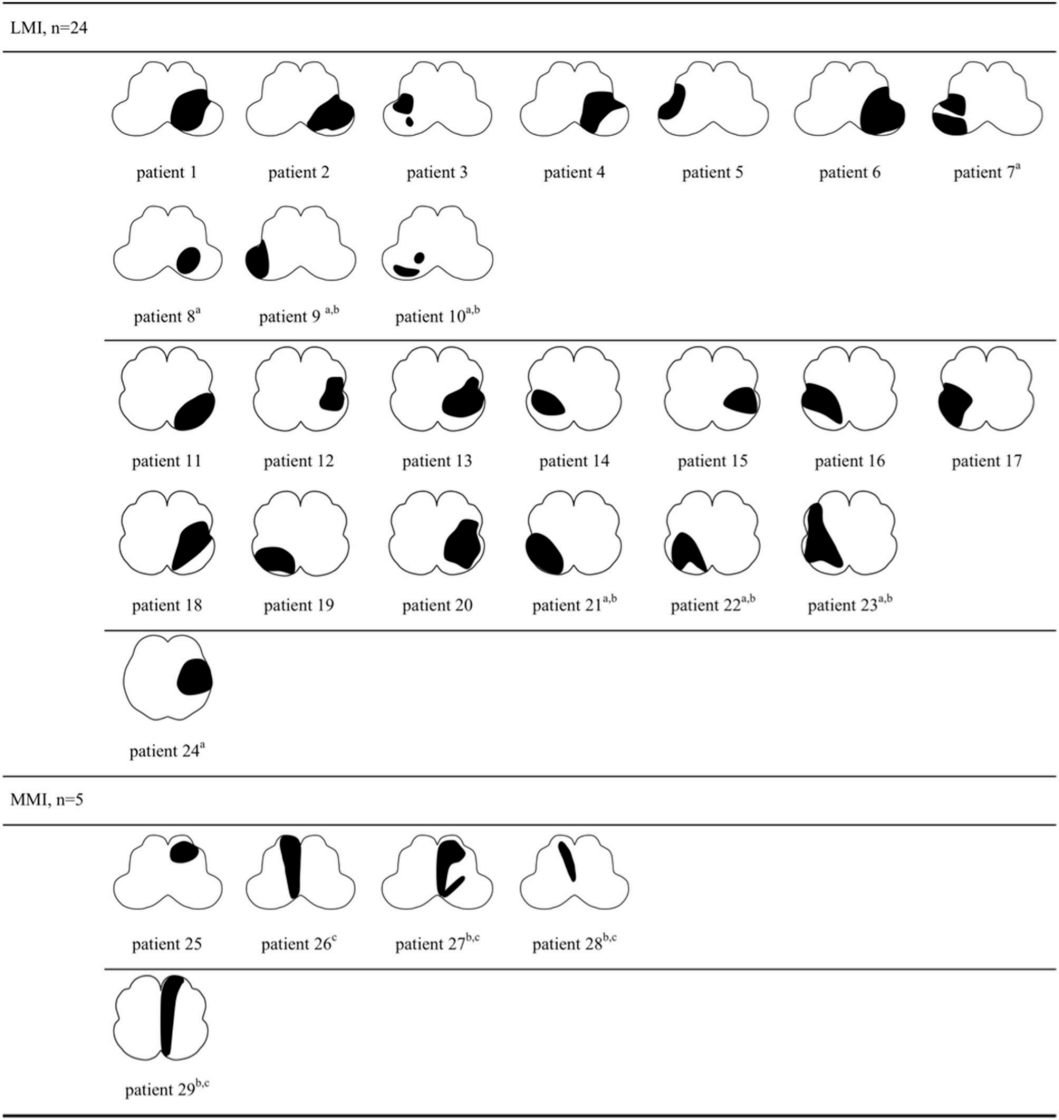
Lesion topography of medullary infarction caused by large artery atherosclerosis (*n* = 29). a, Concurrent ipsilateral PICA-cerebellar infarcts (patient 7–10, 21, 24, all with LMI). b, Co-infarcts of other territories (patient 9, 10, 21–23 with LMI, patient 27–29 with MMI). c, MMI with dorsal extension (patient 26–29). LMI, lateral medullary infarction; MMI, medial medullary infarction.

**Figure 3:**
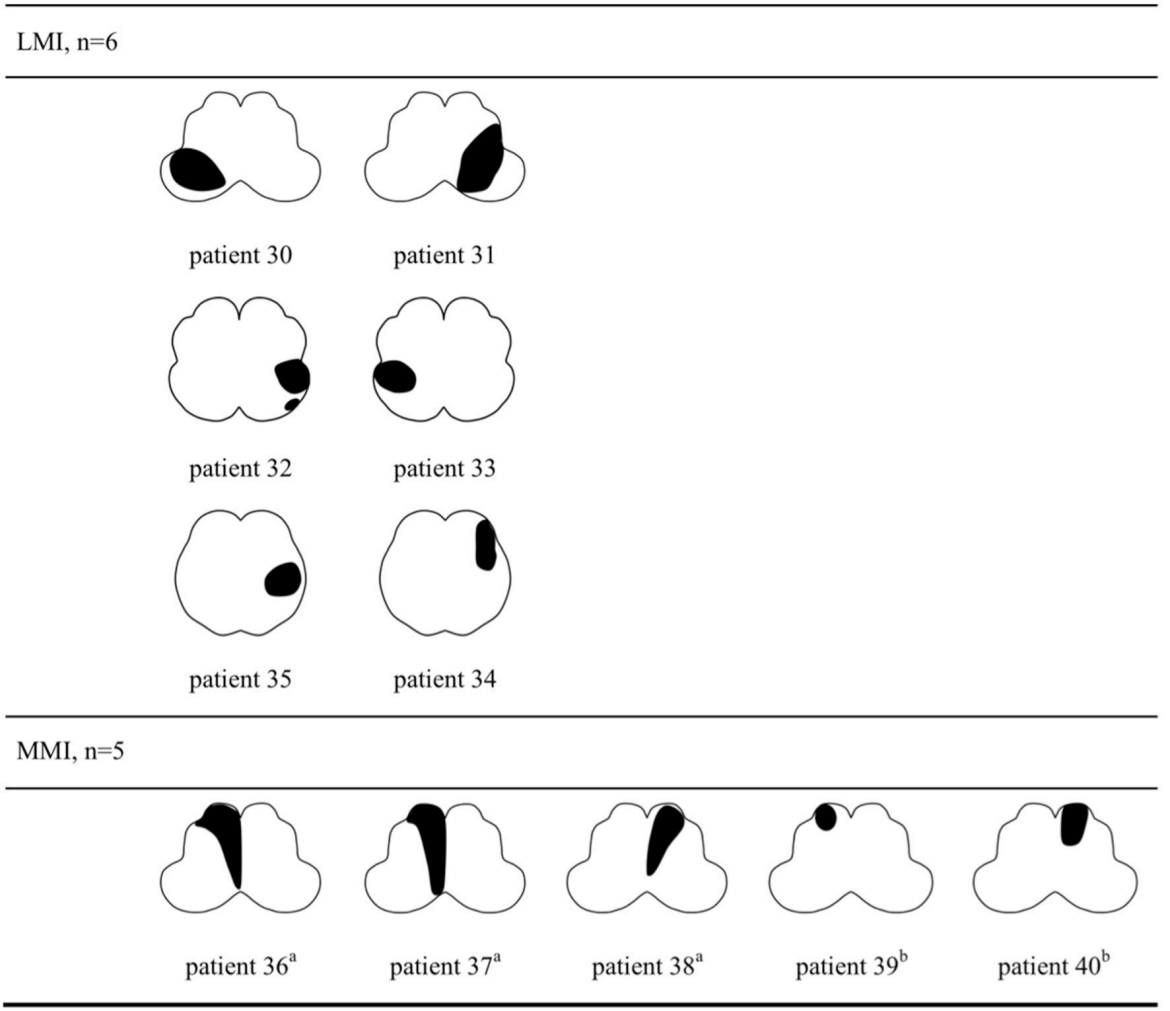
Lesion topography of medullary infarction caused by small vessel occlusion (*n* = 11). a, MMI with dorsal extension. b, Ventral MMI with continuous medial infarcts in lower pons (patient 39 and 40). LMI, lateral medullary infarction; MMI, medial medullary infarction.

MI was identified on DWI and further evaluated on T2 and T1-weighted images by two readers. As was illustrated in Figure 1, the medulla was divided as rostral, middle and caudal according to previous studies (28, 29): (1) Rostral medulla, characterized by dorsolateral bulging of the restiform body (Figure 1A); (2) Middle medulla, characterized by the ventral-lateral bulging of the inferior olivary nucleus (Figure 1B); (3) Caudal medulla, characterized by its round shape with closed fourth ventricle (Figure 1C). Horizontally, MI was classified as lateral and medial (shown in gray color) infarctions. In the case of MMI, lesions were further categorized as ventral (involving the pyramid), middle (involving the medial lemniscus) or dorsal (involving the medial longitudinal fasciculus) as in previous reports (11, 16, 29). Multiple infarcts (co-infarction outside medulla) and lesion diameter were also documented. Maximal lesion diameter in axial T2-weighted images was measured and small infarct was defined as the maximal diameter < 10 mm (18, 30).

### Statistical analysis

Statistical analyses were performed using the IBM SPSS statistics version (SPSS Inc. Chicago, IL, USA). Statistical significance was set at *P* < 0.05. Continuous variables were reported using mean ± standard deviation (SD) for variables with normal distribution and median ± interquartile ranges (IQR) for variables with skewed distribution, they were compared using the student *t* or Mann-Whitney *U-*test. Categorical variables were presented as percentage and compared using the Fisher's exact or Mann-Whitney *U-*tests.

## Results

### Clinical profiles and stroke etiologies

Forty-three patients had medullary infarction (31 male and 12 female) with the mean age of 61 ± 12.1 years. The demographics and risk factors were similar between LMI and MMI (Table 1). The median NIHSS at admission was 4 (IQR 3–5). Patients with MMI had higher admission NIHSS (z = −2.668, *p* = 0.008).

**Table 1:** Clinical profile, stroke etiologies, and infarct pattern of medullary infarction.

	**Total**		**LMI**		**MMI**		*P*-value^*^
	**(*****n*** = **43)**		**(*****n*** = **33)**		**(*****n*** = **10)**		
Male, n (%)	31	(72.1)	24	(72.7)	7	(70.0)	1.000^a^
Age, mean (SD), y	61	(12.1)	59	(12.4)	66	(10.4)	0.143^b^
**Risk Factors**, ***n*** **(%)**							
Hypertension	25	(58.1)	19	(57.6)	6	(60.0)	1.000^a^
Diabetes	11	(25.6)	9	(27.3)	2	(20.0)	1.000^a^
Hyperlipidemia	7	(16.3)	6	(18.2)	1	(10.0)	1.000^a^
Emboligenic heart disease	3	(7.0)	3	(9.1)	0	(0.0)	1.000^a^
Current smoker	14	(32.6)	9	(27.3)	5	(50.0)	0.252^a^
Admission NIHSS, median (IQR)	4	(3–5)	4	(2–5)	8	(4–10)	0.008^c^
**mRS, median (IQR)**							
At discharge	1	(1–2)	1	(1–2)	2	(1–2)	0.580^c^
At follow-up	1	(0–1)	1	(0–1)	1	(1–1)	0.059^c^
Stroke etiology, *n* (%)							0.256^a^
LAA	29	(67.4)	24	(72.7)	5	(50.0)	
SVO	11	(25.6)	6	(18.2)	5	(50.0)	
AD	2	(4.7)	2	(6.1)	0	(0)	
CE	1	(2.3)	1	(3.0)	0	(0)	
Rostro-caudally, *n* (%)							0.009^c^
Rostral	23	(53.5)	14	(42.4)	9	(90.0)	
Middle	17	(39.5)	16	(48.5)	1	(10.0)	
Caudal	3	(7.0)	3	(9.1)	0	(0)	
Multiple infarcts, *n* (%)	12	(27.9)	9	(27.3)	3	(30.0)	1.000^a^
Lesion diameter, mean (SD), mm	7.77	(2.32)	7.26	(2.10)	9.45	(2.32)	0.007^b^
Small infarct, *n* (%)	33	(76.7)	28	(84.8)	5	(50.0)	0.036^a^

* *Comparison between LMI and MMI*.

a *Fisher's exact test*.

b *Student t-test*.

c *Mann-Whitney U-test. LMI, lateral medullary infarction; MMI, medial medullary infarction; LAA, large artery atherosclerosis; SVO, small vessel occlusion; AD, artery dissection; CE, cardiogenic embolism. NIHSS, National Institutes of Health Stroke Scale*.

Large artery atherosclerosis (LAA) was the commonest stroke etiology (29 subjects, 67.4%), followed by small vessel occlusion (SVO, 11 subjects, 25.6%), artery dissection (2 subjects, 4.7%), and cardiogenic embolism (1 subjects, 2.3%). Though emboligenic heart disease was identified in 3 participants, 1 with thrombus in left atrial or ventricle and 2 with atrial fibrillation, the stroke etiology of the latter 2 patients was considered to be LAA concerning the presence of multiple risk factors and marked atherosclerotic changes in relevant arteries. No significant difference of stroke etiologies was observed between LMI and MMI.

### Lesion topography

Of 43 subjects, the rostral, middle and caudal medulla were involved in 23, 17, and 3, respectively. In LMI (*n* = 33), the middle and rostral portion was most frequently affected (16 and 14 subjects, 48.5 and 42.5%). In MMI (*n* = 10), lesions tended to be more rostrally located (9 subjects, 90.0%) and none affected the caudal medulla. 12 (27.9%) patients had multiple infarcts, including the cerebellar, pons, and occipital lobes. The distribution of rostro-caudal topography between LMI and MMI was significantly different (u = 85.000, z = −2.595, *p* = 0.009) while the frequency of multiple infarcts was similar between groups (Table 1). The mean diameter of MI was 7.77 ± 2.32 mm, which was significantly larger in MMI than that in LMI (*t* = −2.819, *p* = 0.007). Two patients had insufficient investigation to determine the presence of VA hypoplasia, for the remaining 41 subjects, VA hypoplasia was seen in 4 (9.8%), 3 at the right side and 1 at the left. Three of four MIs occurred on the contralateral side of VA hypoplasia.

### Lesion-etiology correlation

All 3 medullary infarctions caused by artery dissection (*n* = 2) or cardiogenic embolism (*n* = 1) were in the LMI group, the latter patient also had concurrent PICA-cerebellar infarction. To emphasize the distinction between the infarct pattern caused by large artery atherosclerosis (LAA) and small vessel occlusion (SVO), these 3 participants were excluded, leaving 40 subjects for further analysis.

The infarct pattern caused by LAA and SVO was demonstrated in Table 2. Horizontally, LAA accounted for 24 of 30 lesions in LMI (80.0%) whereas SVO caused 5 of 10 lesions in MMI (50.0%). Rostro-caudally, 28 of 37 middle-rostral lesions were attributed to LAA while 2 of 3 caudal lesions to SVO. All of the 11 subjects with multiple infarcts were caused by LAA. The frequency of small infarct was 23 of 29 in LAA group (79.3%) and 8 of 11 in SVO (72.7%). The lesion distribution, infarct diameter and the frequency of small infarct were not significantly different between LAA and SVO (Table 2).

**Table 2:** Infarct pattern and prognosis of medullary infarction caused by large artery atherosclerosis and small vessel occlusion.

	**LAA**		**SVO**		***P*-value**
	**(*****n*** = **29)**		**(*****n*** = **11)**		
Horizontal, *n* (%)^d^					0.103^a^
LMI	24	(80.0)	6	(20.0)	
MMI	5	(50.0)	5	(50.0)	
Rostro-caudal, *n* (%)^d^					0.695^b^
Rostral	14	(66.7)	7	(33.3)	
Middle	14	(87.5)	2	(12.5)	
Caudal	1	(33.3)	2	(66.7)	
Multiple infarcts, *n* (%)^e^	11	(37.9)	0	(0)	0.019^a^
Lesion diameter, mean (SD), mm	7.92	(1.99)	7.72	(2.96)	0.800^c^
Small infarct, *n* (%)^e^	23	(79.3)	8	(72.7)	0.686^a^
**Favorable Outcome**, ***n*** **(%)**					
At discharge	24	(82.8)	9	(81.8)	1.000^a^
At follow-up^f^	25	(92.6)	11	(100.0)	1.000^a^
Death at follow-up^f^, *n* (%)	2	(7.4)	0	(0)	1.000^a^
Recurrent ischemic events^g^, *n* (%)	2	(8.0)	0	(0)	1.000^a^

a *Fisher's exact test*.

b *Mann-Whitney U-test*.

c *Student t test*.

d *Percentage of rows*.

e *Percentage of columns*.

f Of 38 patients with follow-up no < 3 months (2 patients in LAA group were excluded)

g *Of 36 surviving patients with follow-up no < 3 months. LMI, lateral medullary infarction; MMI, medial medullary infarction; LAA, large artery atherosclerosis; SVO, small vessel occlusion*.

The lesion topography was illustrated in Figures 2, 3. In LAA group (Figure 2), 7 subjects with LMI (patient 7–10, 21–24) and 3 with MMI (patient 27–29) had multiple infarcts. Ipsilateral cerebellar infarctions of PICA territory were seen in 6 subjects, all with LMI (patient 7–10, 21, 24). Co-infarcts of other territories including anterior inferior cerebellar artery, basilar artery, superior cerebellar artery and posterior cerebellar artery were observed in 8 subjects, 5 with LMI (patient 9,10, 21–23) and 3 with MMI (patient 27–29). Four of five MMIs caused by LAA tended to extend from ventral to dorsal (patient 26–29). In SVO group (Figure 3), 2 subjects with ventral MMI also had continuous medial infarcts in lower pons (patient 39 and 40). MMIs with dorsal extension were documented in 3 subjects, however, patient 36 had experienced TIA in the preceding month, patient 37 had previous PICA-cerebellar infarct, the lumen of VA in patient 38 appeared irregular without significant stenosis.

### Follow-up and outcome

At discharge, the median mRS was 1 (IQR 1–2). Thirty-six of 43 subjects (83.7%) were functionally independent (mRS 0–2). For the follow-up analysis, 2 patients in LAA group were excluded because the period was < 3 months. At last follow-up (median 17 months, IQR 15–18 months), the median mRS was 1 (IQR 0–1), 39 of 41 subjects (95.1%) were functionally independent. During follow-up, 2 patients died and 2 experienced recurrent ischemic events, all in LAA group. The rate of favorable outcome, recurrent ischemic events or death at follow-up was similar in patients who received structured or usual care for secondary stroke prevention.

The prognosis of medullary infarction caused by LAA and SVO was displayed in Table 2, the frequencies of favorable outcome, death and recurrent ischemic events were not significantly different between groups.

## Discussion

The major findings of this study are: (1) Ventral MMI with continuous medial pontine infarction is more likely attributed to small vessel occlusion, whereas MMI with particular dorsal extension is more often caused by large artery disease, which warrants further investigations. (2) Though the prognosis of MI is generally favorable, the risk of recurrent ischemic events or death is probably higher in large artery atherosclerosis than that in small vessel occlusion.

We attempted to correlate the lesion pattern of medullary infarction with stroke etiology in our series. With limited cases, the following patterns were considered to be relevant to small vessel occlusion (SVO) or large artery atherosclerosis (LAA): (1) Two patients with ventral MMI had continuous medial pontine infarctions and categorized as SVO based on normal angiography and absence of embolism. To our knowledge, similar result has never been observed previously. This novel finding would probably add to the spectrum of characteristic lesion topography of medullary infarction that indicates SVO rather than LAA. (2) MMI with particular dorsal extension was documented in 7 patients, four in LAA group and 3 in SVO. Of note, though the latter 3 patients were categorized as SVO according to TOAST classification, the presence of preceding TIA, previous PICA-cerebellar infarct and irregular lumen of distal VA raises the possibility that these lesions might be attributed to branch atheromatous disease without significant stenosis/occlusion in parent artery (31), which were also mentioned in previous studies (16–18). These results provide additional evidence that LAA, not SVO, may be associated with ventral-dorsal lesion in MMI. Interestingly, we have also found that infarcts caused by artery dissection or cardiogenic embolism (*n* = 3) and co-occurrence of PICA-cerebellar infarct (*n* = 7) are only detected in LMI. The pattern of ischemia is determined by mixed factors including etiology, mechanism, vascular variation, and collateral status. As PICA is one of the supplier of lateral medulla and also the largest branch of VA which usually arises almost in the direct line of the parent trunk, it is predisposed anatomically to occlusion by emboli (5). This shared mechanism of embolism involved in LAA, artery dissection, and cardiogenic embolism appears to be correlated with the dominance of LMI than MMI. However, co-occurrence of medullary and inferior cerebellar infarction is uncommon because the major supply of lateral medulla is through the branches of distal VA, rather than PICA, thus occlusions in VA without involvement of PICA may spare the cerebellum (32). Moreover, there are extensive collaterals among PICA, superior cerebellar artery, and contralateral arteries, which would provide prompt and effective compensation once the PICA occluded (33). The medial medulla is supplied by ASA at caudal-middle level and by direct penetrators of VA at rostral level. The size and other anatomical features of ASA suggest its immunity from embolism, very numerous reinforcements received throughout its course and anastomosis with the posterior spinal system tend to prevent any widespread occlusion (34). This may explain the rarity of caudal MMI as well as the critical role of small vessel occlusion in rostral MMI.

Few studies compare the long-term prognosis of medullary infarction caused by LAA and SVO. One single-center study with 3-months follow-up (14) demonstrated that 49 of 65 patients with MI (75.4%) had favorable outcome (mRS 0–1), three of 34 subjects in LAA group (8.8%) and none in SVO experienced recurrent ischemic stroke. Another study with long-term follow-up of MMI revealed that 41 of 71 subjects (57.7%) had favorable outcome (mRS 0–2) and recurrent strokes occurred in 11 subjects (15.5%), without comparison between LAA and SVO. In the present study, over 90.0% patients in both LAA and SVO group were functionally independent at the median follow-up of 17 months. However, death (2 of 38 subjects) and recurrent ischemic events (2 of 36 subjects) were documented only in LAA group. For the 2 deceased patients, one had single small LMI in middle medulla with previous infarcts in left occipital lobe and pontine (patient 18) while the other had large MMI in upper medulla with co-infarcts scattered in contralateral occipital lobe and cerebellar (patient 27), both of which indicating arterial embolism due to LAA. More aggressive preventive strategy should be administered in this entity.

MI is rare in our series (3.8%) with a male dominance in their 60s, in line with previous findings (1, 4, 19, 35). The neurological deficits measured by NIHSS are moderate while some disrupting symptoms (e.g., gait instability, hearing loss, vertigo, and nystagmus) might be overlooked. Most patients are functional independent at long-term follow-up. In our series, lateral and middle-rostral medulla is more frequently affected, which agrees favorably with other reports (2, 11, 15, 36). Similarity also exists in that the frequent involvement of VA does not necessarily result in co-infarction of medulla and cerebellum (33). As for the stroke etiology, LAA is the commonest etiology, while artery dissection and cardiogenic embolism are less common and SVO is more frequently identified in our series. Compared with LMI, the neurological deficits of MMI are more severe, mainly due to the involvement of pyramid tracts. The remaining clinical profiles including age, gender, risk factors are similar between LMI and MMI. The middle-rostral lesions dominate LMI whereas MMI would involve more rostral portions. The prevalence of MI (3.8%) and ratio of LMI and MMI (3:1) in our series are similar to previous reports (2, 4, 19). However, some other studies reported higher prevalence of MI (1) or MMI (16). The main reason probably lies in the high rate of false-negative stroke, which accounts for ~1/5 to 1/4 of MI (14, 19), especially small infarct involving the medial or caudal medulla. It is reported that 3 of 8 medial (37%) (19) and 7 of 18 (39%) (17) dorsal medullary infarctions might appear normal in first DWI. Of note, some of these medical centers had applied thin-sliced MRI or repeated MRI several days after the incident in suspected subjects, which was not required in our protocol.

In the present study, four of 41 patients (9.8%) had VA hypoplasia. Interestingly, three were contralateral to side of medullary infarction. Several observational studies suggest that VA hypoplasia may contribute to posterior circulation ischemia (26, 27, 37, 38), however, our cases indicate that the vascular lesion of the dominant VA, not the hypoplasia vessel, may more likely result in ischemic events. It is possible that compensatory mechanisms exist in the case of vascular hypoplasia, making it a harmless anatomic variant.

The methodological strengths of this study include the multicenter prospective design of the SMART study, even though this subgroup analysis is retrospective. Second, we focus on the lesion-etiology correlation, with particular emphasis on the distinct lesion pattern of medullary infarction caused by LAA and SVO. There are some limitations, including a small sample size and insufficient evaluation of large arteries by DSA and/or high-resolution MRI (HRMRI). The majority of angiography performed in our series is MRA, which is unable to detect the arterial wall abnormality and branch artery disease (such as PICA). It is possible that the branch atheromatous disease without significant stenosis/occlusion in parent artery would be missed, thus falsely categorized as SVO rather than LAA. However, this “mistake” would not bother the current stroke management because the mainstay therapy of isolated branch atheromatous disease is medication whereas endovascular or surgical treatment might be the option for selected patients with LAA. Considering the trending utilization of non-invasive technology such as HRMRI, it is promising that these issues would be addressed in future studies.

## Conclusions

With limited cases, the results of the present study demonstrate that medullary infarction has distinct lesion topography depending on various etiologies and mechanisms. To further establish the lesion pattern of MI and validate its correlation with stroke etiology and mechanism, future investigation with larger sample size is of great importance, which may improve stroke management.

## Author contributions

JN, BP, and YH initiated the study. YH drafted the manuscript. JN and BP revised the manuscript substantially and approved its final version. LZ, MY, YZ, and LC participated in the interpretation of data and revised the manuscript. All authors read and approved the manuscript.

### Conflict of interest statement

The authors declare that the research was conducted in the absence of any commercial or financial relationships that could be construed as a potential conflict of interest.
